# Diverse functions of matrix metalloproteinases during fibrosis

**DOI:** 10.1242/dmm.012062

**Published:** 2014-02

**Authors:** Matthew Giannandrea, William C. Parks

**Affiliations:** 1 Center for Lung Biology, University of Washington, Seattle, WA 98122, USA.; 2 Women’s Guild Lung Institute, Cedars-Sinai Medical Center, Los Angeles, CA 90048, USA.

**Keywords:** Fibrosis, Extracellular matrix, Collagen, Metalloproteinase, TIMP

## Abstract

Fibrosis – a debilitating condition that can occur in most organs – is characterized by excess deposition of a collagen-rich extracellular matrix (ECM). At first sight, the activities of proteinases that can degrade matrix, such as matrix metalloproteinases (MMPs), might be expected to be under-expressed in fibrosis or, if present, could function to resolve the excess matrix. However, as we review here, some MMPs are indeed anti-fibrotic, whereas others can have pro-fibrotic functions. MMPs modulate a range of biological processes, especially processes related to immunity and tissue repair and/or remodeling. Although we do not yet know precisely how MMPs function during fibrosis – that is, the protein substrate or substrates that an individual MMP acts on to effect a specific process – experiments in mouse models demonstrate that MMP-dependent functions during fibrosis are not limited to effects on ECM turnover. Rather, data from diverse models indicate that these proteinases influence cellular activities as varied as proliferation and survival, gene expression, and multiple aspects of inflammation that, in turn, impact outcomes related to fibrosis.

## Introduction

Fibrosis is a disease state that typically results from dysfunctional wound healing in response to tissue injury. Normal repair results in the near re-establishment of baseline levels and organization of extracellular matrix (ECM), whereas, in fibrosis, ECM production is excessive and uncontrolled. The main constituents of fibrotic lesions are interstitial collagens, such as type I and III, and excessive deposition of these durable fibers can result in disruption of proper tissue structure and function. Fibrotic lesions occur in most major organs and result in substantial morbidity and mortality.

Both the normal and fibrotic response to tissue injury, as caused by trauma, environmental insults, infection, surgery, etc., involves multiple cellular and molecular players, and many of the mechanistic elements of these processes have been reviewed elsewhere (Duffield et al., 2013; Wynn, 2008; Wynn and Ramalingam, 2012). One class of molecules that is thought to be important in the maintenance of the ECM and processes of tissue repair are matrix metalloproteinases (MMPs). Here, we discuss data demonstrating or suggesting varied roles for specific MMPs in fibrosis of liver, lung and kidney derived from experimental models that utilize genetic deletion or transgenic overexpression of specific MMPs.

## MMPs: more than matrix-degrading enzymes

MMPs are a family of extracellular endopeptidases defined by conserved pro-domains and catalytic domains (Ra and Parks, 2007). As a family, mammalian MMPs comprise 25 members that are either secreted or membrane-bound enzymes. Although MMPs have long been considered to be primarily responsible for turnover and degradation of ECM substrates, they are now recognized as being responsible for mediating crucial functions in a variety of processes, particularly related to immunity and repair, such as cell migration, leukocyte activation, antimicrobial defense, chemokine processing and more (Gill and Parks, 2008; Manicone and McGuire, 2008; Parks et al., 2004). Thus, although limited to secreted, extracellular or membrane proteins, the spectrum of MMP substrates goes well beyond ECM components. Furthermore, studies with gene-targeted mice have demonstrated rather convincingly that individual MMPs perform functions that do not overlap with the role of other MMPs (Gill et al., 2010b). Although degradation studies with candidate substrates show that isolated MMPs have much redundancy *in vitro*, *in vivo* the functions of individual MMPs are limited, specific and apparently unique. An important caveat of *in vitro* degradation assays is that they show what an MMP can do, not what it does do *in vivo*. Indeed, as has been widely discussed (Egeblad and Werb, 2002; Gill and Parks, 2008; McCawley and Matrisian, 2001; Parks et al., 2004), findings from *in vitro* studies on MMP catalysis have been poor predicators of their physiologic function. The diverse roles that MMPs seem to carry out in fibrotic conditions underscore this conundrum.

## Unexpected roles for MMPs in fibrosis

If MMPs only act to degrade ECM and if ECM accumulates in fibrosis, then fibrosis would be, in part, a condition of deficient proteolysis. However, as we discuss in this Review, MMPs have both inhibitory and stimulatory roles in fibrosis (Table 1). Whereas some MMPs do indeed function to reduce fibrosis, others promote it. And, when identified, the mechanism of an MMP function in fibrosis is usually unrelated to direct proteolysis of ECM.

**Table 1.**
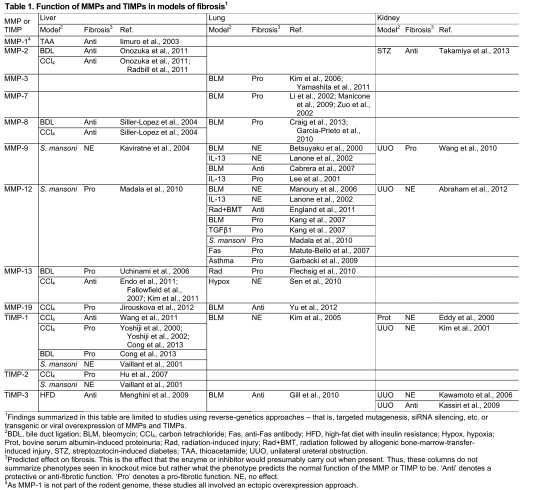
Function of MMPs and TIMPs in models of fibrosis^1^

1 Findings summarized in this table are limited to studies using reverse-genetics approaches – that is, targeted mutagenesis, siRNA silencing, etc. or transgenic or viral overexpression of MMPs and TIMPs.

2 BDL, bile duct ligation; BLM, bleomycin; CCl_4_, carbon tetrachloride; Fas, anti-Fas antibody; HFD, high-fat diet with insulin resistance; Hypox, hypoxia; Prot, bovine serum albumin-induced proteinuria; Rad, radiation-induced injury; Rad+BMT, radiation followed by allogenic bone-marrow-transfer-induced injury, STZ, streptozotocin-induced diabetes; TAA, thioacetamide; UUO, unilateral ureteral obstruction.

3 Predicted effect on fibrosis. This is the effect that the enzyme or inhibitor would presumably carry out when present. Thus, these columns do not summarize phenotypes seen in knockout mice but rather what the phenotype predicts the normal function of the MMP or TIMP to be. ‘Anti’ denotes a protective or anti-fibrotic function. ‘Pro’ denotes a pro-fibrotic function. NE, no effect.

4 As MMP-1 is not part of the rodent genome, these studies all involved an ectopic overexpression approach.

Although we focus on function, control of MMP catalysis is also relevant to understanding how these proteases impact fibrosis or, for that matter, any disease or repair process. Compared with normal repair, the activity of an effector MMP might be over- or underrepresented in fibrosis, and changes in overall activity can be shaped by two mechanisms.

First, biosynthesis of the MMP might be altered. Expression of most MMPs, including essentially all members discussed here, seems to be controlled at the level of transcription. Hence, over- or downregulation of an MMP would indicate that the effectors controlling gene expression in a given cell type are also altered.

The second mechanism(s) that shape MMP activity are various controls over enzyme activity. These mechanisms include zymogen activation and compartmentalization of the active proteinase processes, and these concepts have been discussed in detail elsewhere (Hadler-Olsen et al., 2011; Murphy and Nagase, 2011; Ra and Parks, 2007; Sela-Passwell et al., 2010). In addition, the catalytic activity of MMPs can be silenced by the four tissue inhibitors of metalloproteinases – TIMP-1, TIMP-2, TIMP-3 and TIMP-4 – among other mechanisms, such as endocytosis, oxidative modifications and other inhibitors (Ra and Parks, 2007). The proteinase-antiproteinase paradigm states that net MMP proteolysis in a tissue is the sum of total active MMPs minus inhibition by TIMPs. TIMPs inhibit MMPs with a precise stoichiometry (typically 1:1); however, there are only four TIMP proteins but over 20 MMPs, as well as many other metalloproteinases that can be blocked by TIMPs. Therefore, demonstrating a metalloproteinase imbalance – that is, an excess of metalloproteinase activity over what can be blocked by the available inhibitors – in a tissue homogenate cannot be achieved by examining a few members. However, changes in total metalloproteinase activity can be demonstrated (Gill et al., 2010a; Hu et al., 2010). In discussing MMP function in models of fibrosis, we will also include relevant data on TIMPs.

Development of fibrosis, as well as nonpathogenic normal scarring in response to wounding, can be broken down into four phases (irrespective of affected organ): (1) the precipitating injury or insult, (2) an inflammatory response, (3) activation and differentiation of resident fibroblasts and pericytes or other structural resident cells into α-smooth muscle actin (αSMA)-positive, interstitial-collagen-producing myofibroblasts (Box 1), and (4) tissue remodeling and resolution, i.e. the clearance of fibrotic matrix by extracellular proteolysis and/or endocytosis. In chronic fibrotic conditions, such as liver cirrhosis, idiopathic pulmonary fibrosis and chronic kidney disease, the resolution phase is defective. αSMA, an intracellular contractile protein, is an oft-used marker of myofibroblasts and as a surrogate marker of active collagen deposition and collagen-producing cells in fibrosis. However, it is not clear whether all αSMA-positive cells deposit collagen or if all collagen-producing cells are αSMA-positive.

**Box 1. EMT and αSMA: conflicting models**

Although studies in various organs of animal models have suggested that fibrogenic myofibroblasts can derive from epithelial cells that undergo epithelial-to-mesenchymal transition (EMT) and migrate into the interstitial compartment (Iwano et al., 2002; Kim et al., 2006; LeBleu et al., 2013; Rowe et al., 2011), these findings have been questioned by compelling lineage-tracing studies and other means (Chu et al., 2011; Duffield et al., 2005; Hung et al., 2013; Kriz et al., 2011; Lin et al., 2008; Rock et al., 2011). It is our opinion that the prevailing data indicate that fibrogenic myofibroblasts in lung, kidney and liver arise from resident interstitial cells (i.e. fibroblasts and pericytes). Myofibroblasts are typically identified by expression of α-smooth muscle actin (αSMA), an intracellular contractile protein. αSMA is an oft-used surrogate marker of active collagen deposition and collagen-producing cells in fibrosis. However, it is not clear whether all αSMA-positive cells deposit collagen or if all collagen-producing cells are αSMA-positive.

Where and how do MMPs function in these phases? Although MMPs are thought to have primary roles in the maintenance of ECM, they also are involved in regulating the activity of a range of immunoregulatory molecules. MMPs can contribute to the degree of initial injury and repair, to the onset and resolution of inflammation, to the activation and de-activation of myofibroblasts, and to the deposition and breakdown of ECM. In other words, MMPs are involved in both augmenting and attenuating many processes that impact fibrosis. Related to the relevance of MMPs in fibrotic conditions, some groups have proposed that circulating levels of specific MMPs or possible MMP degradation products are reliable biomarkers of active fibrosis (Leeming et al., 2011; Rosas et al., 2008; Veidal et al., 2011).

**Fig. 1.**
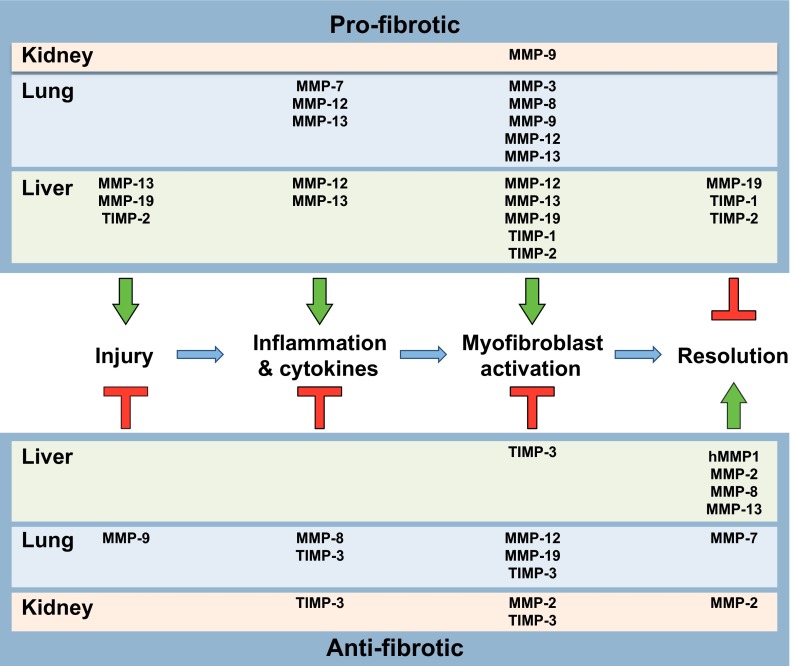
**Model of the roles of MMPs and TIMPs in fibrosis**. In this schematic, the pathogenesis of mammalian fibrosis is broken down into four sequential events, operating over a timescale of weeks to months in animal models and longer in human conditions. First, injury or some sort of damage initiates tissue remodeling responses. Second, an influx of inflammatory cells and the expression of cytokines by both resident and immune cells contribute to the third stage – activation of myofibroblasts and excessive deposition of extracellular collagen, giving the typical appearance of fibrosis. Finally, during the resolution phase, myofibroblasts undergo apoptosis or deactivation, and excess collagen and other matrix components are degraded and cleared. The clearance process can be delayed or inhibited, leading to chronic fibrotic disease, tissue malfunction, organ stress and, ultimately, end-stage disease. By either promoting (arrows) or antagonizing (T-shapes) these processes, specific MMPs and TIMPs have net pro-fibrotic (upper section) or anti-fibrotic (lower section) properties. (See Table 1 or text for references.)

Below, we discuss experimental data demonstrating or suggesting functional roles, both protective and detrimental, for specific MMPs in liver, lung and kidney fibrosis. Although there are many mechanisms for generating fibroses common among these organs, notably including the stable activation of myofibroblasts from resident interstitial cells and common immune features, it is important to note that the roles for specific MMPs are not necessarily the same among different organ systems (Table 1; Fig. 1).

## Roles of MMPs in liver fibrosis

Chronic damage to hepatocytes by viral infection (hepatitis B or C) or excessive alcohol consumption can lead to liver cirrhosis, which consists of extensive fibrosis, loss of metabolic function and, eventually, death. Liver cirrhosis is a prevalent disease, responsible for over 1 million deaths worldwide in 2010 (Lozano et al., 2012). The common experimental models for studying liver fibrosis involve the administration of a chemical toxin, such as carbon tetrachloride (CCl_4_) or thioacetamide (TAA), to cause acute hepatocellular injury or by bile-duct ligation (BDL) to cause cholestasis-induced liver fibrosis. To cause chronic injury, CCl_4_ is given over a period of weeks to months, resulting in significant perivascular fibrosis (Sen et al., 2010). A major collagen-producing cell in the liver is the hepatic stellate cell (HSC) (Kisseleva and Brenner, 2007). When injured, HSCs differentiate into myofibroblast-like cells expressing αSMA and interstitial collagens. By contrast, clearance of fibrotic scar tissue is associated with HSC apoptosis or de-differentiation of HSCs (Kisseleva and Brenner, 2007).

### MMP-1

A potential therapeutic strategy for fibrosis would be to induce expression of a collagenase that would degrade the fibrotic scar tissue (Iimuro and Brenner, 2008), and this approach was demonstrated in a rat model of TAA-induced liver fibrosis (Iimuro et al., 2003), as well as in models of heart and muscle fibrosis (Foronjy et al., 2008; Kaar et al., 2008). After the establishment of fibrosis, animals were infected with an adenovirus expressing human MMP-1 (also known as collagenase-1), which is not present in the rodent genome (Balbín et al., 2001). In addition to reducing the fibrosis, overexpression of human MMP-1 increased hepatocyte proliferation yet led to a moderate amount of diverse tissue damage (Iimuro et al., 2003). These data are potentially important because there are no treatments on the market that reverse fibrosis.

### MMP-2

CCl_4_ and BDL studies with *Mmp2*^−/−^ mice suggest that MMP-2 (also known as gelatinase-A) is anti-fibrotic (Onozuka et al., 2011; Radbill et al., 2011); that is, fibrosis was greater in knockout mice compared with wild-type controls. However, MMP-2 does not seem to be anti-fibrotic by directly remodeling ECM and, indeed, *in vitro* studies have established that MMP-2 is not a collagenase (Seltzre and Eisen, 1999; Tam et al., 2004). Notably, Radbill and colleagues reported that livers from wild-type and *Mmp2*^−/−^ CCl_4_-treated mice have similar levels of collagenase activity and αSMA (Radbill et al., 2011). These data suggest that breakdown of collagen and myofibroblast activation do not seem to be affected by MMP-2. Furthermore, proteins that regulate MMP-2 activity, such as MMP-14 (also known as MT1-MMP) and TIMP-2 (Hernandez-Barrantes et al., 2000), were also produced at similar levels in livers from wild-type and *Mmp2*^−/−^ CCl_4_-treated mice. What was dysregulated by loss of MMP-2 was expression of type I collagen. Compared with wild-type mice, expression of collagen α1 (ColIα1), the principal chain of type I collagen, was elevated in the fibrotic livers of *Mmp2*^−/−^ mice. Furthermore, cell culture data demonstrate that exogenous MMP-2 suppresses collagen expression in HSC-like cells (Radbill et al., 2011). Collectively, these data suggest that anti-fibrotic effects of MMP-2 predominately influence the degree of collagen deposition during activation of liver HSCs. It is unclear how MMP-2 mediates this effect, but a likely mechanism would be proteolysis of a surface protein that results in altered outside-in signaling.

Similarly, Onozuka and colleagues reported increased expression of ColIα1 in CCl_4_-injured *Mmp2*^−/−^ mice, as well as elevated levels of αSMA, TGFβ1, MMP-14, PDGF and TIMP-1 (Onozuka et al., 2011). Although these data are not inconsistent with a role for MMP-2 in influencing collagen expression, they do suggest that other MMP-2-dependent pathways contribute to liver fibrosis. It is important to note that Onozuka and colleagues used a longer treatment regime (12 weeks) compared with the 6-week regime used by Radbill and colleagues (Radbill et al., 2011). Therefore, the overexpression of collagen by HSCs in the absence of MMP-2 might drive increased HSC activation and subsequent dysregulation of other MMPs and TIMPs. Furthermore, increased ColIα1 expression was also observed in the BDL model (Onozuka et al., 2011), suggesting that MMP-2 plays a similar role in different forms of liver fibrosis.

### MMP-8

*In vitro* assays demonstrate that MMP-8 (also known as collagenase-2 or neutrophil collagenase) can cleave interstitial collagens types I and III, and, thus, expression of this proteinase would be expected to be anti-fibrotic by means of promoting collagen breakdown and deactivation of HSCs during the resolution phase. Similar to the MMP-1 transduction studies described above, in both rat CCl_4_ and BDL models of fibrosis, adenoviral transduction of MMP-8 into liver after the establishment of fibrosis resulted in reduction of fibrotic tissue (Siller-López et al., 2004). Mechanistically, it appears that MMP-8 is acting primarily by breaking down collagen, because expression of mRNAs for collagens I and III and transforming growth factor β1 (TGFβ1), an HSC activator and well-established profibrotic cytokine, was unchanged by addition of MMP-8. In addition to degradation of the fibrotic scar tissue, transduction of MMP-8 resulted in enhanced hepatocyte growth factor (HGF) expression and hepatocyte proliferation. These data are consistent with the tissue repair paradigm by which breakdown of interstitial collagens results in apoptosis of HSCs and promotes entry into the hepatocyte cell cycle. Siller-López and colleagues reported increased MMP-2 expression in CCl_4_-treated rats expressing transduced MMP-8, whereas there was no change in MMP-2 expression in their BDL model (Siller-López et al., 2004). Based on the *Mmp2*^−/−^ mouse studies (Onozuka et al., 2011; Radbill et al., 2011), it is possible that MMP-2 contributes to reducing fibrosis by downregulating collagen expression. How MMP-8 expression translates to increased MMP-2 expression in one injury model (CCl_4_) but not another (BDL) remains unclear. Because MMP-8 can also cleave the chemokines CXCL8 and LIX, resulting in enhanced chemoattractant activities (Balbín et al., 2003; Van den Steen et al., 2000; Van den Steen et al., 2003), inflammatory roles for this MMP in fibrosis need to be considered.

### MMP-9, MMP-12 and MMP-13

MMP-12 (macrophage metalloelastase) and MMP-13 (collagenase-3) are macrophage proteinases (although both are expressed by other cell types, too) that are upregulated in response to the T helper 2 (Th2) cytokines IL-4 and IL-13, but not Th1 cytokines, specifically IFNγ and TNFα. Although MMP-12 deficiency has yet to be tested in a CCl_4_ model of liver injury, one study has evaluated fibrosis induction by the parasite *Schistosoma mansoni* in *Mmp12*^−/−^ mice (Madala et al., 2010). Infection by *S. mansoni* eggs results in a granuloma-associated fibrosis that, in contrast to injury-induced fibrosis, appears to be driven by the Th2 cytokine IL-13, with little-to-no role for the traditional fibrotic stimulant TGFβ1 (Chiaramonte et al., 2001; Kaviratne et al., 2004; Wynn, 2004). Loss of MMP-12 production resulted in a modest reduction in granuloma-associated fibrosis at the peak of the response, but similar pathology was also observed at later time points, indicating that MMP-12 plays a modest role in generating fibrosis.

Mechanistically, Madala and colleagues demonstrated that the loss of MMP-12 results in an overall increase in metalloproteinase activity in the liver associated with elevated levels of MMP-2, MMP-9 (gelatinase-B) and MMP-13 (Madala et al., 2010). Although MMP-2 can suppress type I collagen expression (Radbill et al., 2011), loss of MMP-12 does not seem to affect ColIα1 transcription. In addition, it is likely that elevated MMP-9 does not impact the altered fibrotic response in *Mmp12*^−/−^ mice. A report from the same group using *Mmp9*^−/−^ mice infected with *S. mansoni* demonstrated no change in fibrosis (Kaviratne et al., 2004). Therefore, Madala and colleagues focused on MMP-13 and proposed a model in which IL-13-driven MMP-12 production promotes the expression of the decoy IL-13Rα2, which acts as a negative regulator of MMP-13. The loss of MMP-12 thus results in reduced production of the IL-13 decoy receptor with subsequent increase in IL-13 signaling and elevated MMP-13-mediated collagenase activity. Although data from *Mmp13*^−/−^ mice are lacking, the authors demonstrated first that MMP-13 collagenolytic activity is increased and IL-13Rα2 expression reduced in *Mmp12*^−/−^ livers, second that MMP-13 colocalizes with macrophages and myofibroblasts within granulomas, and third that the loss of IL-13Rα2 results in increased MMP-13 expression. Together, these data suggest a model whereby MMP-12 indirectly promotes fibrosis via suppression of MMP-13, an anti-fibrotic MMP.

Much like the MMP-1 studies mentioned above, other groups have used gene therapy technology to assess whether MMP-13 can reduce scarring in CCl_4_-injured liver (Endo et al., 2011; Kim et al., 2011). The idea that MMP-13 is anti-fibrotic is supported by observations of persistent fibrosis in *Mmp13*^−/−^ mice (Fallowfield et al., 2007). But does MMP-13 indeed ameliorate fibrosis by clearing deposited collagen? Overexpression studies by Endo and colleagues suggest that MMP-13 promotes tissue remodeling by converting latent pro-HGF to the active cytokine, thus promoting hepatocyte proliferation and repair (Endo et al., 2011). Interestingly, elevated levels of MMP-2 and MMP-9 were detected in fibrotic livers overexpressing MMP-13 (Endo et al., 2011). However, it is unlikely that the protective effect of overexpressing MMP-13 is due to MMP-2 and MMP-9 acting on collagen directly (Aimes and Quigley, 1995; Seltzre and Eisen, 1999). Contrary to its apparent role in CCl_4_-mediated fibrosis, in the BDL model of injury, loss of MMP-13 had an overall protective effect consisting of reduced cytokine production, myofibroblast activation and collagen expression during the acute phase, and reduced fibrotic areas during the chronic phase (Uchinami et al., 2006). Because pathogenesis differs between the CCl_4_ and BDL models, it is not surprising that certain proteins can have opposing roles in these liver injury models. However, certain common responses stand out. For instance, the loss of MMP-13 in the BDL model results in reduced MMP-2 and MMP-9 expression, which is consistent with the overexpression studies that induced these two MMPs. Although not apparent in the CCl_4_ studies, data from Uchinami and colleagues (Uchinami et al., 2006) is supportive of published work suggesting that MMPs – particularly MMP-2 – contribute to the proliferation of stellate cells (Benyon et al., 1999).

### MMP-19

MMP-19 can degrade various ECM proteins *in vitro* and, thus, could be predicted to act in an anti-fibrotic manner by promoting breakdown of the ECM. However, liver fibrosis is reduced in *Mmp19*^−/−^ mice compared with wild-type controls (Jirouskova et al., 2012), suggesting that MMP-19 is pro-fibrotic. Consistent with the diverse functionality of MMPs, liver injury (as measured by serum alanine aminotransferase levels) is reduced in *Mmp19*^−/−^ mice. Supporting the idea that MMP-19 promotes hepatocellular injury, Jirouskova and colleagues demonstrated impaired TGFβ1 signaling and reduced apoptosis in *Mmp19*^−/−^ mice in response to CCl_4_ injury. Loss of MMP-19 led to increases in the expression levels of MMP-2 and MMP-13, although not all changes were statistically significant (Jirouskova et al., 2012). Despite the lack of significance, the trends observed are consistent with the aforementioned anti-fibrotic roles of these MMPs. Furthermore, Jirouskova et al. uncovered a potential dual role for MMP-19. Although *Mmp19*^−/−^ animals have reduced injury and fibrosis, they appear to resolve fibrosis at a rate slower than do wild-type controls. In wild-type mice, an increase in MMP-19 expression correlates with the resolution of fibrosis (Jirouskova et al., 2012). Thus, MMP-19 seems to be pro-fibrotic in the early phases post-injury, and anti-fibrotic during resolution.

### TIMPs

Liver-specific overexpression of TIMP-1 leads to more severe fibrosis without a significant effect on collagen synthesis (Yoshiji et al., 2000; Yoshiji et al., 2002). Although these data support the parsimonious explanation that TIMP-1 promotes fibrosis by inhibiting the activity of metallocollagenases, such as MMP-13 and MMP-14, increased fibrosis is also seen in *Timp1*^−/−^ mice (Wang et al., 2011). Conversely, mice with siRNA knockdown of TIMP-2 (Hu et al., 2007) following CCl_4_ injury showed reduced HSC activation and collagen deposition, suggesting a pro-fibrotic function. These roles during chemical injury do not appear to apply during Th2-mediated injury because deficiency of TIMP-1 or TIMP-2 does not seem to affect liver fibrosis in response to *S. mansoni* infection (Vaillant et al., 2001). Although the role of TIMP-3 has not been assessed in the liver fibrosis models discussed, perisinusoidal fibrosis is seen in *Timp3*^−/−^ mice with nonalcoholic fatty liver disease (Menghini et al., 2009). Hence, as a group, the TIMPs seem to have divergent roles during liver fibrosis and do not appear to function strictly by blocking the matrix-degrading or, more specifically, the collagenolytic activity of metalloproteinases.

## Role of MMPs in pulmonary fibrosis

Fibrotic diseases of the lung represent diverse etiologies, resulting in similar pathophysiology. Pulmonary fibrosis can result from exposure to allergens, infections, chemicals or radiation. Most often, the source of injury is unknown, hence leading to a diagnosis of idiopathic pulmonary fibrosis (IPF). IPF affects upwards of 200,000 people in the USA, with about 50,000 new cases each year (Raghu et al., 2006) and mortality occurring usually within 3 to 5 years of diagnosis. Current therapeutics have focused predominantly on limiting pulmonary inflammation, with little success. Experimental models, potential cellular etiological mechanisms and therapeutic approaches have been extensively reviewed (Datta et al., 2011; Mouratis and Aidinis, 2011; Wynn, 2011; Wynn and Ramalingam, 2012).

The most frequently used model for studying pulmonary fibrosis involves the use of the chemotherapeutic agent bleomycin. Administration of a single dose of bleomycin to the lungs results in extensive epithelial injury, permeability and a profound inflammatory response, followed by development of interstitial fibrosis. The fibrotic stage lasts for about 3 to 4 weeks and resolves thereafter. Although this model has been criticized for not mimicking non-resolving IPF in humans, it has been the backbone of many potential targets that are currently in clinical trial. As an alternative, a mouse model utilizing repeated doses of bleomycin has been proposed to more closely replicate pathological features of IPF in humans (Degryse et al., 2010). Next, we discuss experimental data examining functional roles for specific MMPs in lung fibrosis.

### MMP-3

MMP-3 (stromelysin-1) can process latent TGFβ1 to its active form, predicting that this MMP is pro-fibrotic. Indeed, as reported by Yamashita and colleagues, fibrosis develops in lungs following ectopic adenoviral expression of MMP-3, and bleomycin-induced fibrosis is dramatically reduced in *Mmp3*^−/−^ mice (Yamashita et al., 2011). However, unexpectedly, levels of active TGFβ1 were comparable between wild-type and *Mmp3*^−/−^ mice. The authors suggested that MMP-3 promotes fibrosis by inducing epithelial cells to undergo an epithelial-mesenchymal transition (EMT) to generate cells that are myofibroblast-like in function. Although it has been suggested that EMT is a source of myofibroblasts in lung (Kim et al., 2006) and other organs, lineage-tracing studies do not support the view that any type-I-collagen-producing cells in bleomycin-induced fibrosis arise from the epithelium (Rock et al., 2011) (Box 1). Thus, how epithelial-derived MMP-3 functions to promote fibrosis remains an open question.

### MMP-7

A potential role for MMP-7 (matrilysin) in pulmonary fibrosis was initially identified through gene expression analysis of human fibrotic lung samples and was found to be highly upregulated compared with its expression in healthy tissue (Zuo et al., 2002). Although *Mmp7*^−/−^ mice are protected from bleomycin-induced acute lung injury (Li et al., 2002; Manicone et al., 2009), they develop extensive fibrosis, although slightly reduced from that in wild-type mice (Li et al., 2002; Zuo et al., 2002), suggesting a pro-fibrotic role for this protease. MMP-7 cleaves syndecan-1, which carries the chemokine CXCL1 as cargo on its glycosaminoglycan chains, from injured lung epithelial cells, and that release of the syndecan-1–CXCL1 complexes is required for the transepithelial infiltration of neutrophils (Li et al., 2002). Demonstrating that MMP-7-dependent neutrophil infiltration contributes to development of fibrosis, Manicone and colleagues found that recovery of transepithelial neutrophil influx in *Mmp7*^−/−^ mice – through instillation of the bacterial chemotactic peptide *N*-formylmethionyl-leucyl-phenylalanine) – restored fibrosis to wild-type levels (Manicone et al., 2009). Thus, it is possible that MMP-7 promotes fibrosis indirectly by facilitating neutrophil influx and activation and, in turn, enhanced epithelial damage that leads to more fibrosis. However, MMP-7 also facilitates epithelial repair (Chen et al., 2008; Chen and Parks, 2009; Dunsmore et al., 1998; McGuire et al., 2003), also through shedding of syndecan-1 (Chen et al., 2009), probably balancing to some degree any added injury from allowing neutrophils to transmigrate.

In addition to a pro-fibrotic consequence due to an acute process, Manicone and colleagues proposed that MMP-7 also functions to help resolve fibrosis (Manicone et al., 2009). MMP-7 sheds syndecan-1 during the first 5–7 days post-bleomycin treatment, yet the expression of MMP-7 persists and increases for up to 25 days (Li et al., 2002). MMP-7 begins to shed the intact 80-kDa ectodomain of E-cadherin at about 10 days post bleomycin treatment (McGuire et al., 2003). The E-cadherin ectodomain can ligate α_E_β_7_ integrin (CD103), which is expressed on a subset of T cells and CD11c^hi^ dendritic cells. Manicone and colleagues found that fibrosis is elevated and persists in mice lacking the α_E_ subunit (*Itgae*^−/−^), which only pairs with the β_7_ integrin subunit. They proposed that shedding of E-cadherin acts to home and activate immunosuppressive dendritic cells that aid in the resolution of fibrosis (Manicone et al., 2009). Overall, the findings with MMP-7 indicate that it serves both pro- and anti-fibrotic functions and that these opposing functions are separated in time. Early on, MMP-7 functions in the innate response by facilitating neutrophil influx and activation, leading to collateral epithelial damage and an enhanced fibrotic environment. Then later, epithelial-derived MMP-7 promotes resolution by attracting an influx of immunosuppressive leukocytes.

### MMP-8

Two studies have reported reduced fibrosis in *Mmp8*^−/−^ mice, indicating that this proteinase has pro-fibrotic functions (Craig et al., 2013; García-Prieto et al., 2010). These findings are opposed to observations in the liver, where overexpression of MMP-8 led to improved resolution of fibrosis (Siller-López et al., 2004). The mechanisms explaining how MMP-8 promotes pulmonary fibrosis differs between the two studies. García-Prieto and colleagues observed a reduction of active TGFβ1 that they link to the loss of MMP-8-mediated inactivation of IL-10 (García-Prieto et al., 2010). Hence, their model predicts that MMP-8 blocks IL-10 signals, leading to enhanced fibroblast activation and collagen production. This idea, however, is counter to data demonstrating induction of lung fibrosis by overexpression of IL-10 (Sun et al., 2011). By contrast, other investigators did not observe any differences in levels of active TGFβ1 or IL-10 in *Mmp8*^−/−^ mice with bleomycin-induced fibrosis, although they did find increased levels of CXCL10 (IP-10) and CCL3 (MIP-1α) (Craig et al., 2013). Furthermore, when they crossed *Mmp8*^−/−^ mice with *Cxcl10*^−/−^ or *Ccl3*^−/−^ mice, fibrosis was restored to wild-type levels (Craig et al., 2013). Although these data are consistent with the proposed pro-fibrotic role for MMP-8, further data are needed to uncover the underlying mechanism. However, the data presented do suggest that MMP-8 influences multiple processes by modulating cellular inflammation, cytokine inactivation and/or fibroblast activation.

### MMP-9

Like MMP-3, MMP-9 can activate latent TGFβ1 (Yu and Stamenkovic, 2000). This activity, along with expression levels that correlate positively with experimental fibrosis (Murthy et al., 2010), suggests a pro-fibrotic role for MMP-9. However, similar to observations in a model of liver fibrosis (Kaviratne et al., 2004), bleomycin-induced pulmonary fibrosis does not differ between wild-type and *Mmp9*^−/−^ mice (Betsuyaku et al., 2000). By contrast, bleomycin-induced fibrosis is reduced in transgenic mice constitutively expressing human MMP-9 in macrophages under control of the promoter for scavenger receptor class A (Cabrera et al., 2007). Cabrera and colleagues linked MMP-9-mediated attenuation of fibrosis to reduced insulin growth factor binding protein-3 (IGFBP-3), which normally sequesters IGFs and prevents cell growth. They hypothesized that with reduced IGFBP-3 there is increased recovery from injury and less cell death that would potentially drive fibrosis.

Overexpression of an MMP might lead to unexpected or unnatural functions (see Box 2). It could be that excess MMP-9 acts directly on lung ECM, as was proposed for the destructive consequence of overexpression of MMP-9 in a model of atherosclerosis (Gough et al., 2006). In a model of IL-13-driven fibrosis, lack of MMP-9 results in reduced fibrosis, which was linked to activation of TGFβ1 (Lee et al., 2001). It is interesting to note that the IL-13 lung-fibrosis model is dependent on TGFβ1, whereas other IL-13-dependent models of fibrosis (e.g. *S. mansoni*) are not (Kaviratne et al., 2004).

**Box 2. Caveats of overexpressing MMPs to uncover function**

As discussed in the text, functions for some MMPs, such as MMP-1 and MMP-9, in fibrosis have been suggested from studies that involved overexpression – typically by viral transduction – of the enzyme. There are two important caveats associated with this type of experimental strategy, caveats that are relevant for many types of proteins. First, when overexpressed, an MMP might act on non-physiological substrates, mediating misleading responses. Owing to excess levels of the enzyme, the proteinase:substrate stoichiometry becomes skewed in favor of the enzyme. Because MMPs lack inherent substrate specificity (unlike, for example, coagulation cascade proteinases), their activity *in vivo* is likely to be limited to specific proteins by allosteric interactions that confine the proteinase to pericellular niches and, in turn, substrates. An excess of a given MMP would allow some portion of the enzyme produced to escape these regulatory mechanisms. The concept that MMP activity is confined by interactions with other extracellular macromolecules has been discussed elsewhere (Hadler-Olsen et al., 2011; Murphy and Nagase, 2011; Ra and Parks, 2007; Sela-Passwell et al., 2010; Tocchi and Parks, 2013). The second caveat with overexpression is that – unless cell-specific promoters are used – the enzyme can be produced ectopically. That is, the MMP might end up being made by a cell type that does not make the enzyme naturally, leading to the strong potential of acting on non-physiological substrates. In addition, the cell in which the MMP is ectopically expressed could lack the allosteric control mechanisms discussed under the first caveat, thereby adding the potential artefactual responses. Overexpression approaches demonstrate what an MMP *can* do, not what it *does* do. On the positive side, overexpression might point to potential therapeutic strategies, as suggested by the overexpression of MMP-1 in mouse models of fibrosis.

It seems that, as appears to be the case for many MMPs, the jury is still out on the nature of the role for MMP-9 in fibrosis. Thus, MMP-9 expression resulted in less fibrosis in one study (Cabrera et al., 2007), whereas in another it was pro-fibrotic (Lee et al., 2001), while yet others concluded it had no role (Betsuyaku et al., 2000; Kaviratne et al., 2004). In models of allergic lung inflammation, MMP-9 has been shown to have various functions related to chemokine levels and leukocyte influx (Corry et al., 2004; Greenlee et al., 2006), and other studies have demonstrated an ability to cleave and alter the activity of various chemokines (Van den Steen et al., 2000; Van den Steen et al., 2003). Clearly, assessing immunomodulatory roles for MMP-9 in fibrosis needs to be examined in further depth.

### MMP-12

The role of MMP-12 during lung fibrosis has been studied in a number of different injury models, most of which attribute its role as being pro-fibrotic (Garbacki et al., 2009; Kang et al., 2007; Madala et al., 2010; Matute-Bello et al., 2007). With the bleomycin model, there are two conflicting studies. Whereas one group observed reduced fibrosis in *Mmp12*^−/−^ animals compared with wild-type controls (Kang et al., 2007), another reported no difference in fibrosis between the two genotypes (Manoury et al., 2006). However, the discrepancy between these two reports might be explained by the time point at which fibrosis was assessed. Manoury and colleagues assessed fibrosis at 2 weeks post-bleomycin (Manoury et al., 2006), whereas Kang and colleagues looked later, at 3 weeks (Kang et al., 2007). After 2 weeks of bleomycin treatment, excessive collagen deposition is still barely evident (increased collagen expression can be seen by day 7). Hence, an MMP-12-driven effect might not manifest until fibrosis is more advanced. Kang and colleagues also demonstrated a similar pro-fibrotic role for MMP-12 in transgenic mice overexpressing TGFβ1. The authors stated – but did not show – that inflammation did not differ between *Mmp12*^−/−^ and wild-type mice, suggesting that MMP-12 influences downstream processes such as fibroblast activation or collagen production (Kang et al., 2007).

Pro-fibrotic roles for MMP-12 were also found in models of pulmonary fibrosis generated by anti-Fas antibody (Jo2), which targets the tumor necrosis factor receptor, and infection with *S. mansoni*. As with bleomycin studies, MMP-12 deficiency did not affect inflammation during anti-Fas-antibody-mediated injury (Matute-Bello et al., 2007). However, gene expression analysis of Jo2-treated *Mmp12*^−/−^ mice indicated reduced expression of genes implicated in fibroblast migration and proliferation, suggesting that MMP-12 contributes to fibroblast activation. Observations from animals infected with *S. mansoni* suggest that MMP-12 promotes fibrosis indirectly by suppressing expression of other anti-fibrotic MMPs (Madala et al., 2010).

Contrary to previous studies suggesting a pro-fibrotic role, two contrasting studies, one using a fibrosis model with transgenic mice expressing IL-13 (Lanone et al., 2002) and the other a model involving radiation injury followed by bone-marrow transfer (so-called ‘RAD+BMT’) (England et al., 2011), observed either no effect or an anti-fibrotic effect of MMP-12, respectively. Although a direct mechanistic link to fibrosis is unclear, both studies reported altered expression of other MMPs in *Mmp12*^−/−^ mice, similar to that found in the *S. mansoni* model (Madala et al., 2010).

### MMP-13

Two conflicting studies addressed the role of MMP-13 during injury-induced lung fibrosis. One study by Flechsig and colleagues observed that, after radiation-induced injury, *Mmp13*^−/−^ mice had reduced fibrosis coupled with decreased inflammation (Flechsig et al., 2010). However, Sen et al., using a hyperoxia injury model, reported that the extent of fibrosis did not differ between *Mmp13*^−/−^ and wild-type mice. They did, however, find that inflammation was elevated in *Mmp13*^−/−^ mice, and *in vitro* data demonstrated that MMP-13 can cleave and inactivate monocyte chemokine CCL2 (MCP-1) (Sen et al., 2010). Other studies have demonstrated that MMP-13-generated cleavage of specific chemokines – CCL7 and CXCL12 – leads to reduced chemotactic activity (McQuibban et al., 2001; McQuibban et al., 2002). Many MMPs, including MMP-13, are known to process various chemokines, resulting in either enhanced, diminished or altered activity (Gill and Parks, 2008; McQuibban et al., 2000; Parks et al., 2004; Van Lint and Libert, 2007). Because cellular inflammation is believed to be a key element in the development of fibrosis, the regulation of leukocyte chemotaxis by MMP-mediated chemokine modification could be a significant contributing factor to disease severity.

### MMP-19

MMP-19 was identified as a potential target by a gene expression array that showed that MMP-19 was strongly upregulated in hyperplastic epithelial cells of IPF patients compared with epithelial cells of normal lung tissue (Yu et al., 2012). However, evaluation of fibrosis in *Mmp19*^−/−^ mice showed increased hydroxyproline and αSMA staining compared with the levels in wild-type animals. Thus, the overexpression of MMP-19 in IPF patient lungs could be indicative of a reparative response. Yu and colleagues proposed that MMP-19 exerts an anti-fibrotic effect by inducing prostaglandin G/H synthase 2 (PTGS2; also known as COX2), which generates prostaglandin E2 (PGE2). PGE2 suppresses fibrosis by inhibiting fibroblast migration, proliferation, collagen synthesis and differentiation into myofibroblasts (Huang et al., 2009; Kolodsick et al., 2003; White et al., 2005). This hypothesis was supported by the loss of PTGS2 expression in bleomycin-treated *Mmp19*^−/−^ mice and the induction of PTGS2 by A549 cells overexpressing recombinant MMP-19.

### TIMPs

TIMPs have not been extensively studied in models of lung fibrosis. In contrast to its potential protective role in liver fibrosis (Wang et al., 2011), TIMP-1 does not impact the development or extent of lung fibrosis in response to bleomycin (Kim et al., 2005). However, it has been reported that fibrosis is elevated and persists in bleomycin-injured *Timp3*^−/−^ lungs (Gill et al., 2010a). Interestingly, enhanced fibrosis occurred in *Timp3*^−/−^ lungs despite an overall increase in metalloproteinase activity (Gill et al., 2010a), further questioning the dogma that MMPs function to degrade or clear ECM. Inflammation is increased in both bleomycin-injured *Timp1*^−/−^ and *Timp3*^−/−^ mice (Gill et al., 2010a; Kim et al., 2005), hence supporting the concept that MMPs function predominately to control immune processes rather than directly promoting or attenuating fibrotic pathways.

## Role of MMPs in kidney fibrosis

Fibrosis is a complication of chronic kidney disease (CKD), which eventually progresses towards end-stage renal disease (ESRD). Like other fibrotic diseases, treatments are limited. For those with severe disease, treatment involves dialysis and kidney transplants. In 2009, there were about 90,000 individuals in the USA who died owing to ESRD. This disease is predominately associated with diabetics and the elderly, with ~26% of the population over age 60 having stage 3 CKD (moderately reduced kidney function). Cellular and molecular pathways involved in the development of renal fibrosis have been reviewed elsewhere (Carew et al., 2012; Conway and Hughes, 2012; Liu, 2010; Liu, 2011).

Experimental models of renal fibrosis include ureteral obstruction nephropathy, diabetic nephropathy, remnant kidney and chronic allograph nephropathy. Most studies that have addressed MMP function have used the unilateral ureteral obstruction (UUO) model, which involves the ligation of one ureter while leaving the contralateral one patent as a control. Fibrosis develops within the first 7 days, and the kidney reaches end-stage disease after 2 weeks (Chevalier, 2006).

### MMP-2

One study evaluated renal fibrosis in *Mmp2*^−/−^ mice using a diabetic nephropathy model initiated by streptozotocin (STZ), a pancreatic β-cell toxin (Takamiya et al., 2013). Development of fibrosis in this model occurs after 3 months, even though increased activation of fibroblasts is detectable after a couple of weeks (Chow et al., 2004). Similar to increased liver fibrosis in *Mmp2*^−/−^ mice (Onozuka et al., 2011; Radbill et al., 2011), Takamiya and colleagues demonstrated increased collagen deposition and fibroblast activation – as indicated by αSMA production – in diabetic *Mmp2*^−/−^ mice, supporting the view that MMP-2 has an anti-fibrotic role (Takamiya et al., 2013). Furthermore, the levels of *ColIα1* mRNA did not differ between wild-type and *Mmp2*^−/−^ diabetic mice, suggesting that increased fibrosis in the null mice was due, in part, to impaired degradation of collagen. This effect differs from the liver studies, where the loss of MMP-2 impacted *ColIα1* transcription. As mentioned above, MMP-2 is not a collagenase (Seltzre and Eisen, 1999; Tam et al., 2004) and therefore is probably not directly degrading collagen. However, it could help facilitate the clearance of fibrosis by acting on other ECM components. Alternatively, because the expression of other MMPs is altered in *Mmp2*^−/−^ mice (Onozuka et al., 2011), MMP-2 might regulate the severity of renal fibrosis indirectly by promoting expression of other collagen-degrading proteases.

### MMP-9 and MMP-12

As discussed earlier, MMP-9 is a potential activator of latent TGFβ1 and, hence, might be a pro-fibrotic mediator. Indeed, data from a UUO model showed reduced fibrosis in *Mmp9*^−/−^ mice (Wang et al., 2010). However, this reduction in fibrosis was not accompanied by any significant change in active TGFβ1 or TGFβ receptor production between wild-type and *Mmp9*^−/−^ mice. Wang et al. observed a significant decrease in αSMA levels in *Mmp9*^−/−^ mice, suggesting a role for MMP-9 in myofibroblast activation or survival (Wang et al., 2010). Thus, in contrast to its overall pro-fibrotic role in liver and lung, MMP-12 does not seem to impact the severity of fibrosis during UUO (Abraham et al., 2012).

### TIMPs

Two studies evaluating induction of kidney fibrosis by either UUO or protein overload show that the loss of TIMP-1 does not affect disease severity (Eddy et al., 2000; Kim et al., 2001), similar to the lack of an effect on lung fibrosis (Kim et al., 2005) but in contrast to its anti-fibrotic role in liver (Wang et al., 2011). Similar to lung, kidney fibrosis is enhanced in *Timp3*^−/−^ mice subjected to UUO for 2 weeks (Kassiri et al., 2009). The authors suggest that the protective effect of TIMP-3 is due to suppression of TNFα, which mediates renal fibrosis and regulates expression of several MMPs. These data are in contrast to an earlier study that found no difference in renal fibrosis between wild-type and *Timp3*^−/−^ mice after 7 days of UUO (Kawamoto et al., 2006). However, they did detect increases in metalloproteinase activity and TGFβ1 expression in *Timp3*^−/−^ mice, suggesting that, as in lung tissue, TIMP-3 might serve a protective function by suppressing cytokines involved in myofibroblast activation.

## Concluding remarks

MMPs modulate a range of biological processes, especially processes related to immunity, tissue repair and tissue remodeling. Although we do not yet know precisely how MMPs function during fibrosis – that is, the identity of the protein substrate(s) an individual MMP acts on to effect a specific process – we can nevertheless discern important points that could be relevant to future studies and potential targeting of MMPs for clinical therapies. Together, the studies discussed above demonstrate that MMP-dependent functions during fibrosis are not limited to effects on ECM turnover, but rather that these proteinases influence cellular proliferation and survival, gene expression, and multiple aspects of inflammation, and can directly and indirectly influence the activation of myofibroblasts.

As is apparent by the information presented here and summarized in Table 1 and Fig. 1, MMPs have the potential to influence many biological processes during the pathogenesis of tissue fibrosis. To understand better the mechanisms of MMP function during fibrosis, we propose three paramount issues for future experimentation to facilitate more-targeted approaches to specific processes during fibrogenesis. First, we need to identify the cell types expressing relevant MMPs and assess their roles. Although cellular sources of many MMPs are known, expanded use of conditional gene targeting strategies will provide important insights into functional roles during disease states. The establishment of floxed gene repositories, such as the knockout mouse project (KOMP; www.komp.org), facilitates the ability to perform targeted gene-deletion experiments. Second, it is difficult to surmise biological mechanisms without a clear understanding of what protein substrates individual MMPs cleave *in vivo*. Although the identities of many *in vitro* substrates are known, novel methods are needed to experimentally ascertain what targets MMPs are cleaving during injury and repair. Finally, because MMPs can affect production of other family members, future studies need to track production of each of the functionally relevant family members during gene-targeted deletion studies. By evaluating all relevant MMPs together, we will be in a much stronger position to make future well-judged pharmaceutical interventions in these debilitating disorders.
